# Neutral space analysis for a Boolean network model of the fission yeast cell cycle network

**DOI:** 10.1186/0717-6287-47-64

**Published:** 2014-11-25

**Authors:** Gonzalo A Ruz, Tania Timmermann, Javiera Barrera, Eric Goles

**Affiliations:** Facultad de Ingeniería y Ciencias, Universidad Adolfo Ibáñez, Av. Diagonal las Torres 2640, Peñalolén, Santiago, Chile; Center of Applied Ecology and Sustainability (CAPES), Santiago, Chile

**Keywords:** Neutral graph, Boolean networks, Evolution strategy, Fission yeast cell cycle, Attractors

## Abstract

**Background:**

Interactions between genes and their products give rise to complex circuits known as gene regulatory networks (GRN) that enable cells to process information and respond to external stimuli. Several important processes for life, depend of an accurate and context-specific regulation of gene expression, such as the cell cycle, which can be analyzed through its GRN, where deregulation can lead to cancer in animals or a directed regulation could be applied for biotechnological processes using yeast. An approach to study the robustness of GRN is through the neutral space. In this paper, we explore the neutral space of a *Schizosaccharomyces pombe* (fission yeast) cell cycle network through an evolution strategy to generate a neutral graph, composed of Boolean regulatory networks that share the same state sequences of the fission yeast cell cycle.

**Results:**

Through simulations it was found that in the generated neutral graph, the functional networks that are not in the wildtype connected component have in general a Hamming distance more than 3 with the wildtype, and more than 10 between the other disconnected functional networks. Significant differences were found between the functional networks in the connected component of the wildtype network and the rest of the network, not only at a topological level, but also at the state space level, where significant differences in the distribution of the basin of attraction for the *G*_1_ fixed point was found for deterministic updating schemes.

**Conclusions:**

In general, functional networks in the wildtype network connected component, can mutate up to no more than 3 times, then they reach a *point of no return* where the networks leave the connected component of the wildtype. The proposed method to construct a neutral graph is general and can be used to explore the neutral space of other biologically interesting networks, and also formulate new biological hypotheses studying the functional networks in the wildtype network connected component.

## Background

The fate of a cell, and an organism as a whole, is determined by the functioning of a complex cellular machinery. An important part of this machinery, which determines the downstream information, are the gene regulatory networks (GRN). GRN represents the indirect interaction between genes by means of their products (proteins, micro RNA, etc.). Accurate and context-specific regulation of gene expression is essential for all organisms, because vital tasks such as cell differentiation and cell division, homeostasis, apoptosis, metabolism and signal transduction, depend on this regulation. Studies of several processes have been developed through the reconstruction and analysis of gene regulatory networks that underlie these processes. For example, the circadian clock of *Neurospora* and *Arabidopsis thaliana*[1], cell cycle of *Saccharomyces cerevisiae*[2], embryonic segmentation of *Drosophila melanogaster*[3], flower development of *A. thaliana*[4, 5], T-lymphocytes activation of human immune system [6], mammalian cell cycle [7] and the SOS pathway of *E. coli*[8], among others.

The identification of the topology, regulatory nodes of these networks and the hierarchical relationship between them, is essential to understand a particular process (its behavior and dynamic). However, because the molecular interactions within a gene regulatory network are very complex, the functional integration of the network cannot be understood only by intuitive reasoning, therefore, the need to incorporate mathematical models for its study arises. One of the most popular models to describe and analyze the behavior of GRN are Boolean networks, introduced by Stuart Kauffman in 1969 [9]. Boolean networks give a first idea of the qualitative dynamics of a gene regulatory network represented by the temporal evolution of the protein states. In this network, each node represents a gene or protein, that can be either active (node value 1) or inactive (node value 0), and the edges represents regulatory relationships between genes. The dynamics of the network is computed by a set of Boolean functions for each node and an updating scheme (synchronous or asynchronous). For a Boolean network with *n* nodes, there are 2^*n*^ possible states, and given the deterministic nature of this model, the network will converge to steady states, also known as attractors. There are two types of attractors, fixed point, where once the network reaches that state it can never leave it. The other is the limit cycle, where the network returns to a previous state with a certain periodicity. One can also consider a non-deterministic approach, using a fully asynchronous updating scheme, where nodes are updated randomly. Fixed point attractors are invariant to changes or the selection of the updating scheme, nevertheless, limit cycles and the size of basins of attractions may change drastically.

Boolean networks are used to investigate the organizational principles of a network and how this influences their robustness. This mathematical model is commonly reconstructed by three different approaches: (1) based on very detailed knowledge of the process to be modeled (regulatory relations identified in previous publications) [10], (2) from transcriptional analysis of a set of knockouts or mutants [11], and (3) from transcriptional time-series data of wild-type organisms [12]. Inferring the topology of a Boolean network from a set of experimental data involves two main steps: first, the experimental data (gene expression profiles or protein concentrations) must be discretized into maximally informative binary state transitions (0 or 1 values). The second step uses these binary profiles to learn the Boolean network that best captures the Boolean trajectories.

In this paper, we consider the Boolean network model for the fission yeast cell cycle [10]. The cell cycle involves four phases, *G*_1_ (Gap 1), *S* (Synthesis), *G*_2_ (Gap 2) and *M* (Mitosis). In the *G*_1_ phase, cells increase in size. Further, inside this phase there exist an important checkpoint, called “Start point” in yeast. *G*_1_ checkpoint makes the key decision whether the cell should divide (enter to the *S* phase), delay division, or enter a resting stage. This decision will depend on environmental conditions, that increase or not the cell size (final signal). In the *S* phase, DNA replication occurs, in order to duplicate the genetic material. During the gap (*G*_2_) between DNA synthesis and mitosis, the cell will continue to grow. The *G*_2_ checkpoint control mechanism ensures that everything is ready to enter the *M* (mitosis) phase and divide. Finally, when the cell enters into the *M* phase, cell growth stops and cellular energy is focused on the orderly division into two daughter cells. A checkpoint in the middle of the mitosis (metaphase checkpoint) ensures that the cell is ready to complete cell division. After the *M* phase, the cell comes back to the stationary *G*_1_ phase, waiting for the signal for another round of division. It is important to note that the progress through the cell cycle is unidirectional and irreversible, this ensures the proper functioning of the cell cycle.

Given the mathematical representation of the fission yeast cell cycle through a Boolean network, it is of interest to analyze the robustness of the model under perturbation. Previously, the dynamical robustness of this model has been studied in [13]. But not its topological (connectivity) robustness. An approach to study the topological robustness of a GRN, in the sense of modifying the connections (adding or removing edges) of the network, and analyzing the resulting function of the network is through the neutral space introduced in [14]. The neutral space consists of different regulatory networks that share the same function. It can be visualized and analyzed through a neutral graph (also known as a neutral network) developed in [15, 16] that is an undirected graph where each node represents a regulatory network. If two nodes are connected in the neutral space this means that the Hamming distance (number of entries in which two adjacency matrices differ) between the interaction (adjacency) matrix of one network and the other is one. The connectivity of a neutral graph can give an insight of the topological robustness of the regulatory networks. A neutral graph with large connected components (nodes) can be considered of having high robustness whereas a neutral graph with many small connected components (or disconnected) can be considered of having low robustness [15].

In order to construct a neutral graph, we need to construct Boolean regulatory networks that have a certain property in common, this opens the opportunity to use intelligent computational techniques, in particular evolutionary computation and related techniques to construct networks with predefined properties. Evolutionary computation is a subfield of artificial intelligence, inspired from natural evolution, dedicated to solve complex optimization problems. It consists in a group of algorithms, that using the basic elements of biological evolution, explores the solution space through genetic operators like crossover and mutation, and selects the fittest candidate solutions. An example of these algorithms corresponds to Genetic Algorithms (GAs) introduced by John H. Holland in the 1970s [17].

Following this line of research, in [18] simulated annealing, which is based on the annealing treatment of solids consisting in the physical process of heating up a solid until it melts and cooling it down slowly until it crystallizes, changing the properties of the solid, was used to construct Boolean networks with predefined attractors for sequential updating mode only. The swarm intelligence technique called the bees algorithm, which is inspired in the food search strategies used by honeybees, has been formulated to construct Boolean networks with predefined attractors [19] and used to build synthetic networks of the budding yeast cell-cycle in [20], that promote cell proliferation for biotechnological applications. In [21], a reverse engineering technique was applied to the reconstruction of the mammalian cell cycle network using the binary gene expression data generated by the logical model in [7]. This reverse engineering method used an information theoretic approach combined with a modified version of the original bees algorithm. More recently, extensions to that work have been presented in [22] where synthetic networks are constructed that share the same limit cycles (of length seven) and the fixed point of the mammalian cell cycle network.

In this paper we explore the neutral space of the *Schizosaccharomyces pombe* (fission yeast) cell cycle regulatory network. For this, we propose an evolutionary computation algorithm, in particular, an evolution strategy (ES), as a metaheuristic optimization algorithm that uses a mutation operator as its main search strategy (unlike GAs that use crossover and mutation) to generate a neutral graph of Boolean regulatory networks that share the same state sequences of the fission yeast cell cycle. We analyze the resulting neutral graph and compare characteristics of the regulatory networks that appear in the connected component of the original yeast cell cycle network with the networks that are not in the connected component, thus, given us a notion of the robustness of the model.

## Results and discussion

### Proposed evolution strategy (ES) for neutral graph construction

**Figure 1:**
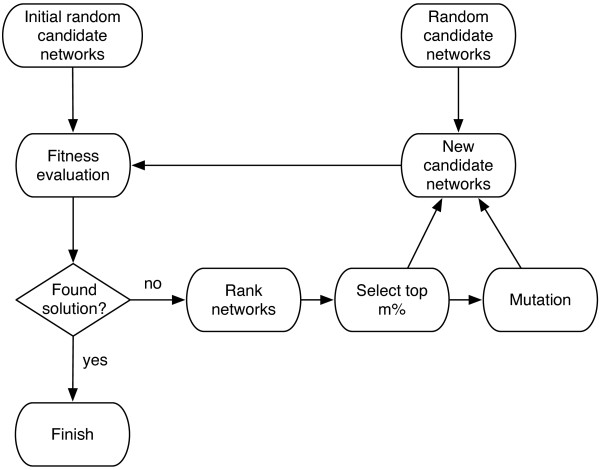
**Evolution strategy (ES) flow chart.** The proposed ES was used to search for functional networks that compose the neutral graph.

As mentioned in the background, a neutral graph is a metagraph (network of networks) where each node represents a regulatory network that produces the same temporal evolution for a set of states out of the 2^*n*^ possible configurations. In this paper, we considered the ten state sequences shown in Table 1. Following the terminology used in [14], regulatory networks that reproduce this temporal evolution will be called *functional* networks, while the original fission yeast cell cycle network will be called *wildtype* network, which is a functional network as well given the previous definition. Although the wildtype network and a functional network will share the same state sequences of Table 1, they do not necessarily share the dynamics produced by the remaining 2^10^-10 states. The connectivity in a neutral graph is given by the Hamming distance of the interaction matrices (adjacency matrices) of the functional networks. Two nodes are connected if the Hamming distance of the respective functional network’s interaction matrices are equal to one, i.e., both matrices differ in only one element. The search in the neutral space for functional networks is huge, which makes it a difficult problem. The search consists in finding the weight matrix elements *w*_*ij*_ and the threshold vector elements *θ*_*i*_ that can replicate the desired state sequences. To carry out the search for functional networks, we propose an evolution strategy (ES) illustrated in the flow chart in Figure 1. Preliminary results using this technique appear in [23]. In what follows we will describe each stage.

**Table 1:** **Temporal evolution of sate vectors defining the fission yeast cell cycle**

Time	Start	SK	Cdc2/Cdc13	Ste9	Rum1	Slp1	Cdc2/Cd13*	Wee1/Mik1	Cdc25	PP	Phase
1	1	0	0	1	1	0	0	1	0	0	START
2	0	1	0	1	1	0	0	1	0	0	*G*_1_
3	0	0	0	0	0	0	0	1	0	0	*G*_1_/*S*
4	0	0	1	0	0	0	0	1	0	0	*G*_2_
5	0	0	1	0	0	0	0	0	1	0	*G*_2_
6	0	0	1	0	0	0	1	0	1	0	*G*_2_/*M*
7	0	0	1	0	0	1	1	0	1	0	*G*_2_/*M*
8	0	0	0	0	0	1	0	0	1	1	*M*
9	0	0	0	1	1	0	0	1	0	1	*M*
10	0	0	0	1	1	0	0	1	0	0	*G*_1_

#### Initial random candidate networks

A user defined parameter *popSize* indicates the size of the initial population. These are generated in the following way. Using as a base the wildtype weight matrix and threshold vector, a new candidate network (solution) is obtained by changing *ngh* times the wildtype adjacency matrix and threshold vector. The parameter *ngh* is selected randomly in the range of [ 1,30], for every new candidate network generated. The wildtype weight matrix is changed using the following rule:

**Rule 1**

1. Select randomly a position (*i*,*j*) in the matrix.
2. If the position contains a non-zero number, then replace by a zero.
3. Else, replace with a value selected randomly from the following set {-2,-1,1,2}.

The wildtype threshold vector is changed using the following rule:

**Rule 2**

1. Select randomly a position *i* in the vector.
2. Replace with a value selected randomly from the following set {-2,-1,-1/2,0,1/2,1,2}.

both rules are repeated *ngh* times.

#### Fitness evaluation

Each candidate network is evaluated in a fitness function defined as follows. The fitness function for the Boolean regulatory network *B*, is computed by the deviation of the network’s output, defined by *o*_*i*_ for each node *i*, and the target value *s*_*i*_ (sequence of the cell cycle) for each node *i*:
1

where *n* is the number of nodes in the network, and 10 is the number of state vector sequences (from Table 1) that the network must contain.

#### Rank networks

Given that the fitness function defines the deviation of the network’s output with respect to the desired target, the ES is formulated to solve a minimization problem, therefore, the candidate networks are ranked from the less deviated to the more deviated.

#### Select top *m*%

A user defined parameter *m* indicates the percentage of the ranked top solutions to be selected.

#### Mutation

Using the top *m*% solutions, (*popSize*-*popSize*×*m%*)/2 new candidate networks are generated using the following rule:

**Rule 3**

1. Select randomly one of the top *m*% solutions.
2. Mutate the selected solution. This is done by applying Rule1 and Rule2 with *ngh*=1.

this is repeated until completing the (*popSize*-*popSize*×*m%*)/2 new candidate networks.

#### Random candidate networks

To complete the *popSize*, the remaining (*popSize*-*popSize*×*m%*)/2 is filled with random candidate networks generated using Rule1 and Rule2 with *ngh* selected randomly in the range of [ 1,30], for every new candidate network generated.

#### New candidate networks

The new population to be evaluated in the fitness function, thus, completing the loop, is composed by the top *m*% solutions + the networks generates by the mutation stage + the networks generated randomly.

### Simulations

The proposed ES was used to construct a neutral graph with functional networks that contain the fission yeast cell cycle state sequences of Table 1. In order to bound the search space, the elements of the weight matrices were constrained to the following integer values: {-2,-1,0,1,2}, and the threshold vectors to: {-2,-1,-1/2,0,1/2,1,2}. Also, *popSize*=20, *m%*=20*%* and max iterations =100.

**Figure 2:**
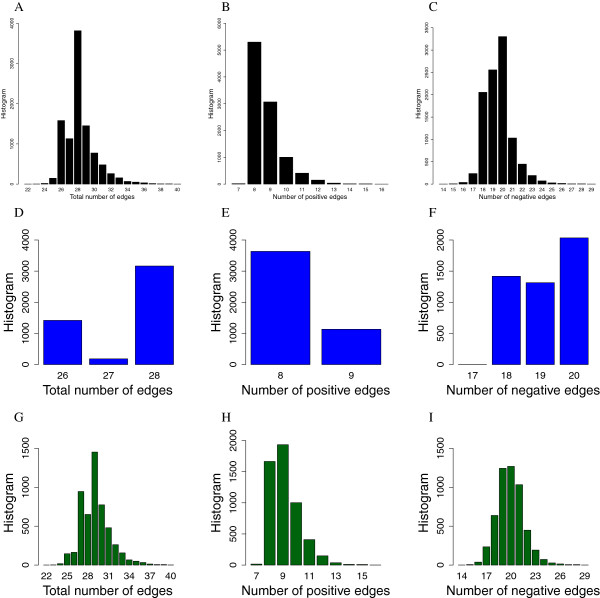
**Histograms of the functional networks topologies of the neutral graph.** **A** Total number of edges; **B** Positive edges; **C** Negative edges; **D** Total number of edges in the wildtype connected component; **E** Positive edges in the wildtype connected component; **F** Negative edges in the wildtype connected component; **G** Total number of edges not in the wildtype connected component; **H** Positive edges not in the wildtype connected component; **I** Negative edges not in the wildtype connected component.

The ES was used to find 10000 functional networks. Histograms of the resulting functional networks topologies are shown in Figure 2, where Figure 2A shows the distribution of the total number of edges (non-zero elements in the weight matrices) in the functional networks, Figure 2B shows the distribution of the number of positives edges and Figure 2C the distribution of the negative edges. If we consider that the wildtype network has a total of 27 edges composed of 8 positive edges and 19 negative edges, we can see from the histograms that in general the functional networks are mostly concentrated in these values but they can also have less or more edges then the wildtype.

The distribution of the edges changes if we separate the functional networks that belong to the wildtype connected component and the functional networks that are not in the connected component. Figure 2D,E and F are histograms from the wildtype connected component, showing that they are more concentrated around the wildtype topology whereas histograms G, H, and I are from functional networks that are not in the connected component, showing a larger dispersion.

**Figure 3:**
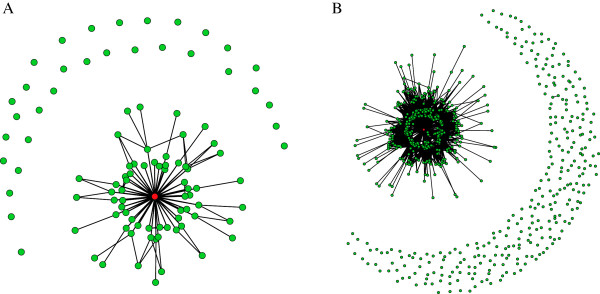
**Neutral graph obtained using the proposed evolution strategy.** (Color online) the red node represents the wildtype network. **A** Neutral graph using 100 functional networks; **B** Neutral graph using 1000 functional networks.

For visualization purposes we sampled 100 and 1000 functional networks from the 10000 found by the ES, to generate the neutral graph in Figure3A and Figure 3B respectively. The wildtype network appears in red (Color online). We notice that for functional networks not in the wildtype connected component, it is rare to see other connected components, in particular, in Figure 3B we see only one additional connected component besides the wildtype one, formed by two functional networks. From the wildtype connected component we notice that functional networks reach the wildtype in no more than 3 steps.

Biological robustness can be analyzed through the topological robustness of the functional networks in the wildtype connected component of the neutral graph. In particular, one can identify which of the edges of these functional networks appear more frequent, and which are less frequent. This can give a notion of the regulatory relations that are required, in a mandatory way in some cases, in order to complete the cell cycle sequence. For this simulation, out of the 10000 functional networks, 330 are in the wildtype connected component. From these networks in the connected component, the positive edge that activates the node *Slp*1 appears in 100% of the networks. This connection to *Slp*1 is necessary to ensure the integrity of the cell cycle, because *slp*1 mutant cells remain arrested in metaphase [24], therefore, the state sequences to complete the fission yeast cell cycle is interrupted in these cells. Another interesting case to point out are the double mutants *rum*1/*wee*1 and *ste*9/*wee*1 which are not viable [25], therefore, all the networks in the wildtype connected component contain the positive edges that activates *Ste*9, *Wee*1 and *Rum*1, whereas, 0.6% of the non-connected component networks do not present the edge that activates *Ste*9. In a similar way, 0.4% of the networks in the non-connected component do not present the edge that activates *Wee*1, and 0.5% do not present the edge that activates *Rum*1. Surprisingly, by analyzing the networks in the wildtype connected component, only one connection appears out of the norm, in 43,9% of the networks, this is a positive edge from *Ste*9 to *Cdc*25. This change in the topology of the wildtype network may allow the possibility to formulate new biological hypotheses which could be tested.

**Figure 4:**
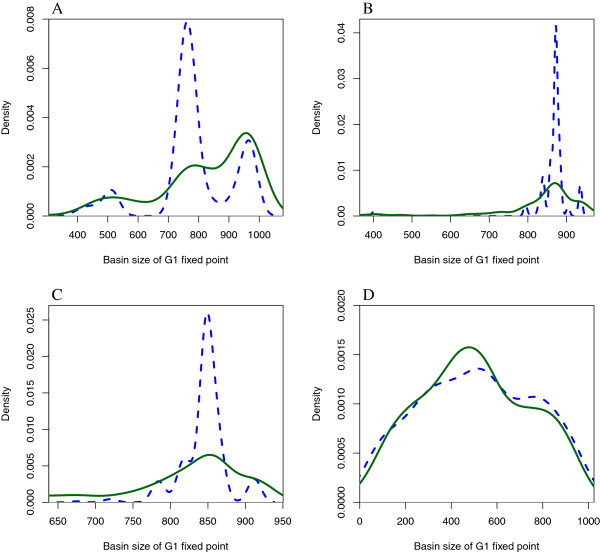
**Density of the basin of attraction for the** ***G***_**1**_ **fixed points of the functional networks in the wildtype component (blue/dashed line) and the rest of the networks (green/solid line).** **A** Using the parallel updating scheme; **B** Using the following block sequential updating scheme (Start,SK,Cdc2/Cdc13)(Ste9,Rum1,Slp1,Cdc2/Cd13 ^∗^)(Wee1/Mik1,Cdc25,PP); **C** Using the following sequential updating scheme (Start)(SK)(Cdc2/Cdc13)(Ste9)(Rum1)(Slp1)(Cdc2/Cd13 ^∗^)(Wee1/Mik1)(Cdc25)(PP); **D** Using the fully asynchronous updating scheme.

**Figure 5:**
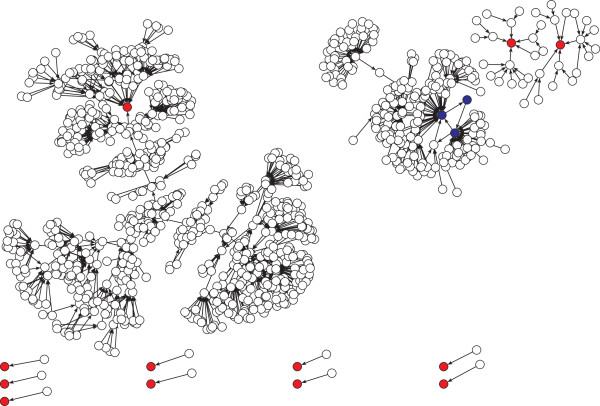
**Sate transition graph of the wildtype network using the parallel updating scheme.** (Color online) The fix point states are represented by red circles and limit cycle states are represented by blue circles.

**Figure 6:**
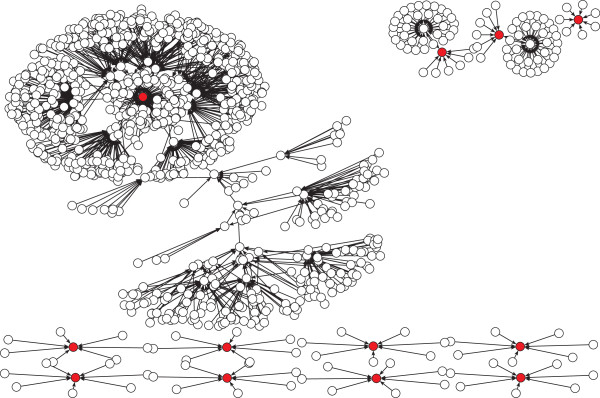
**Sate transition graph of the wildtype network using a block sequential updating scheme.** State transition graph using the following updating scheme (Start,SK,Cdc2/Cdc13)(Ste9,Rum1,Slp1,Cdc2/Cd13 ^∗^)(Wee1/Mik1,Cdc25,PP). (Color online) The fix point states are represented by red circles.

**Figure 7:**
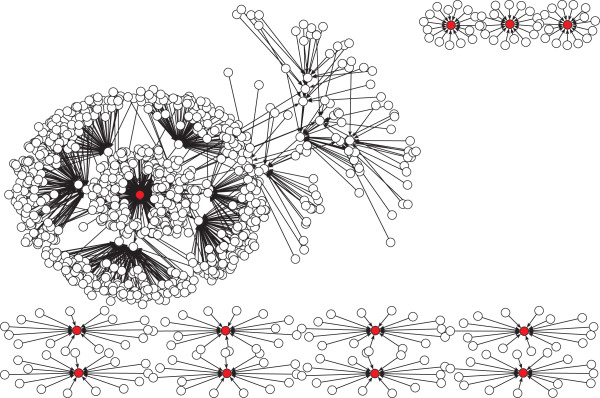
**Sate transition graph of the wildtype network using a sequential updating scheme.** State transition graph using the following updating scheme (Start)(SK)(Cdc2/Cdc13)(Ste9)(Rum1)(Slp1)(Cdc2/Cd13 ^∗^)(Wee1/Mik1)(Cdc25)(PP). (Color online) the fix point states are represented by red circles.

The density of the basin of attraction of the *G*_1_ fixed point of the functional networks in the wildtype connected component (blue/dashed line) and the rest of the networks (green/solid line) appears in Figure 4A for the parallel update, B for a block sequential update, C for a sequential update, and D for the fully asynchronous update. We can appreciate that both densities are quite different, regardless of the updating scheme, except for the fully asynchronous. For the deterministic updating schemes, we notice that while the functional networks in the connected component have a basin of attraction mostly concentrated between 700 ∼900 (the wildtype has a basin of attraction of size 762 using the parallel update), nevertheless, the rest of the networks show a density that stretches out more. For the asynchronous update, we notice that the size of the basin of attraction of the *G*_1_ fixed point does not concentrate in a specific range of values as do the deterministic updating schemes. It seems that each initial state converges to one of the different attractors with equal probability without having a particular preference for the *G*_1_ fixed point as in the other deterministic cases. Finally, Figure 5 shows the state transition graph for the wildtype network using the parallel updating scheme, Figure 6 shows the state transition graph for the wildtype network using a block sequential updating scheme, and Figure 7 shows the state transition graph for the wildtype network using a sequential updating scheme. From these state transition graphs, one can appreciate the large basin of attraction for the *G*_1_ fixed point.

## Conclusions

An evolution strategy was developed to construct a neutral graph of Boolean regulatory networks that share the same state trajectory of the fission yeast cell cycle network [10]. Through simulations it was found that in the generated neutral graph, the functional networks that are not in the wildtype connected component have in general a Hamming distance more than 3 with the wildtype, and more than 10 between the other disconnected functional networks. We found that there are significant differences between the functional networks in the connected component of the wildtype network and the rest of the network, not only at a topological level, but also at the state space level, where significant differences in the distribution of the basin of attraction for the *G*_1_ fixed point was found for deterministic updating schemes, but not for the fully asynchronous updating scheme. From the results one can see that in general functional networks in the wildtype connected component, can mutate up to no more than 3 times, then they reach a *point of no return* where the networks leave the connected component of the wildtype.

Finally, although the proposed evolution strategy was used for the fission yeast cell cycle model, it can be used to construct a neutral graph of other biological models under the Boolean network formalism. Moreover, the neutral space analysis of GRNs, may allow us to formulate new biological hypotheses studying the functional networks in the wildtype connected component, for example, analyzing which edges are in common, yielding a core structure that could explain the preservation of the functionality of the network.

## Methods

### Boolean networks

Let ***x*** be a finite set of *n* variables, ***x***={*x*_1_,…,*x*_*n*_}, with *x*_*i*_∈{0,1} for *i*=1,…,*n*. A Boolean network is a pair (*G*,*F*), where *G*=(**V**,**E**) is a finite directed graph; **V** being the set of *n* nodes and **E** the set of edges. *F* is a Boolean function, *F*:{0,1}^*n*^→{0,1}^*n*^ composed of *n* local functions *f*_*i*_:{0,1}^*n*^→{0,1}. Furthermore, each local function *f*_*i*_ depends only on variables belonging to the neighborhood *V*_*i*_={*j*∈**V**|(*j*,*i*)∈**E**}. The indegree of vertex *i* is |*V*_*i*_|. The updating schemes are repeated periodically, and since the hypercube is a finite set, the dynamics of the network converges to attractors which are fixed points or limit cycles, defined by

- Fixed point: *x*_*i*_(*t*+1)=*x*_*i*_(*t*) for *i*={1,…,*n*}.
- Limit cycle: *x*_*i*_(*t*+*p*)=*x*_*i*_(*t*) for *i*={1,…,*n*}.

where *p*>1 is a positive integer called the period. The set of states that can lead the network to a specific attractor is called the basin of attraction. There are many ways of updating the values of a Boolean network, some examples are:

- Parallel or synchronous mode: where every node is updated at the same time.
- Sequential updating mode: where in every time step, every node is updated in a defined sequence.
- Block-sequential: the set of nodes, for a given sequence, is partitioned into blocks. The nodes in a same block are updated in parallel, but blocks follow each other sequentially.
- Asynchronous deterministic: where in every time step, one node is updated following a defined sequence.
- Fully asynchronous: where in every time step, one node is selected randomly to be updated.

An alternative to working with arbitrary logical gates in each node, is to consider a threshold Boolean network, where updates of each node are computed by
2

with *ω*_*ij*_ the weight of the edge coming from node *j* into the node *i*, and *θ*_*i*_ the activation threshold of node *i*. The weights and thresholds are the network’s parameters.

### Fission yeast cell cycle network

**Figure 8:**
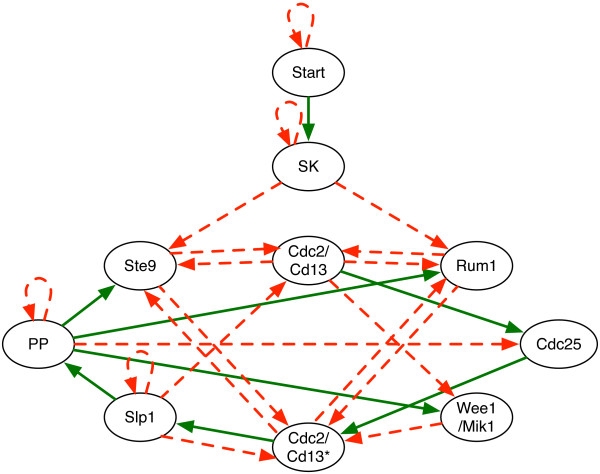
**The fission yeast cell-cycle threshold Boolean network.** Using a similar configuration as [10], (color online) the green/solid edges represent positive weights (activations), the red/dashed edges represent negative weights (inhibitory). The red/dashed loops represent self-degradation.

Let us consider the Boolean network model for the cell cycle of the yeast species *Schizosaccharomyces pombe* (fission yeast) studied in [10] shown in Figure 8.

Using a similar representation as in [10], the green/solid edges are positive weights (activations), the red/dashed edges are negative weights (inhibitory). The red/dashed loops are self-degradation, which are modeled mathematically by a negative weight. The gene updates are computed by a variant of (2), as defined in [10]:
3

**Figure 9:**
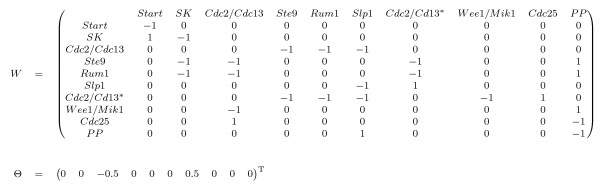
**Parameters for the fission yeast cell-cycle threshold Boolean network.** *W* represents the weight matrix and *Θ* the threshold vector.

The weight matrix and the threshold vector used in (3) to generate the same dynamics exhibited in [10] appears in Figure 9. A complete dynamical study of this model can be found in [13].

In this model, three classes of molecules act: (1) The major role is played by a cyclin-dependent protein kinase complex: Cdc2/Cdc13 with Tyr-15, a residue of Cdc2; (2) positive regulators of the kinase Cdc2/Cdc13: an indicator of the mass of the cell that works as “Start”, “Start kinase” (SK), a group of Cdk/cyclin complexes (Cdc2 with Cig1, Cig2 and Puc1 cyclins), and the phosphatase Cdc25; (3) antagonists of the complex Cdc2/Cdc13: Slp1, Rum1, Ste9, and the phosphatase PP. In the *G*_1_ phase, without the signal of cell size increase, the Cdc2/Cdc13 complex is inactive due to its antagonists. When the cell achieves a certain size, SK becomes active and a new round of cell division will begin by means of the accumulation of the Cdc2/Cdc13 complex. Cdc2/Cdc13 and SK dimers switch off the antagonists Rum1 and Ste9/APC in order to enter into the *S* phase. Moderate level on activity of the Cdc2/Cdc13 complex is enough for entering the *G*_2_ phase but not the mitosis, since proteins kinase Wee1 and Mik1 inhibits the activity of residue Tyr-15 of Cdc2. Then, to achieve the *M* phase, the activity of the Cdc2/Cdc13 complex must increase, and this occurs due to Cdc25 that reverses phosphorylation, removing the inhibiting phosphate group and increasing the activity of the complex. High activity of the Cdc2/Cdc13 complex is represented in the network by a separate node called Cdc2/Cdc13*. An elevated activity level of Cdc2/Cdc13* at the *M* phase, activates Slp1/APC (Anaphase-Promoting complex). Slp1 degrades Cdc13, therefore, the Cdc2/Cdc13* complex is inhibited. At the end of the *M* phase the antagonists of Cdc2/Cdc13 are reset and the cell reaches the *G*_1_ stationary state.

Using the parallel updating scheme, the cell cycle is modeled by starting from an initial state vector at time *t*=1 and then the dynamics of the network produces sequences of state vectors until *t*=10, where the network converges to a fixed point which represent the *G*_1_ phase. Details of the previous sequences are shown in Table 1.
